# Comparative molecular profiles of distinct tumor components in recurrent tentorial meningioma after stereotactic radiosurgery: a case report implicating acquired aggressive alterations associated with WHO grade progression

**DOI:** 10.1007/s10014-025-00524-0

**Published:** 2026-01-06

**Authors:** Takeru Hirata, Yudai Hirano, Motoyuki Umekawa, Satoru Miyawaki, Yuki Shinya, Hirotaka Hasegawa, Yu Sakai, Noritaka Kudo, Daisuke Komura, Hiroto Katoh, Shumpei Ishikawa, Nobuhito Saito

**Affiliations:** 1 Department of Neurosurgery, The University of Tokyo Hospital, 7-3-1 Hongo, Bunkyo-ku, Tokyo, 113-8655 Japan; 2 Department of Neurosurgery, University of Virginia, Charlottesville, VA USA; 3 Department of Pathology, The University of Tokyo Hospital, Bunkyo-ku, Tokyo, Japan; 4 Department of Preventive Medicine, Graduate School of Medicine, The University of Tokyo, Bunkyo-ku, Tokyo, Japan

**Keywords:** Meningioma, Stereotactic radiosurgery, Pathological progression, Somatic mutations, Copy number alteration

## Abstract

Meningiomas, the most common primary benign intracranial tumors, may recur and, in some cases, undergo WHO grade progression, acquiring more aggressive clinical behavior. The molecular mechanisms underlying such progression, particularly following stereotactic radiosurgery (SRS), remain poorly understood. We report a 70-year-old woman with a gradually enlarging tentorial meningioma treated with SRS as primary therapy. The irradiated lesion remained stable on serial MRI for 30 months following treatment. Subsequently, a rapidly enlarging marginal progression was detected posterior to the original tumor site. Surgical resection revealed two distinct pathological components: the anterior, irradiated lesion was a fibrous meningioma whereas the posterior, marginally progressed lesion was diagnosed as an anaplastic meningioma (WHO grade 3). Whole-exome sequencing identified shared *NF2* mutations and losses at 1p and 18p in both components, while the marginally progressed lesion harbored additional high-risk alterations, including homozygous *CDKN2A/B* deletion and loss of the X chromosome. Despite re-irradiation and further surgical resections, the patient succumbed to tumor progression and disseminated disease 8 months after the marginal progression. These findings suggest that a subset of tumor cells may have acquired genetic alterations driving WHO grade progression, ultimately leading to marginal progression after SRS.

## Introduction

Meningiomas are the most common primary benign intracranial extra-axial tumors, which originate from arachnoid cap cells and arise from anywhere in the cranium [1]. In the 2021 World Health Organization (WHO) Classification of Tumors of the Central Nervous System, meningiomas are classified into three grades based primarily on histopathological features, with molecular diagnostics applied in selected cases [2–4]. Surgical resection remains the standard care for patients in good general condition, whereas stereotactic radiosurgery (SRS) is typically employed for small tumors, lesions located in surgically inaccessible or deep-seated regions, patients with poor clinical status, following subtotal resection, or for WHO grade 2–3 lesions [4]. SRS is an established treatment option, with favorable outcomes reported for SRS alone or in combination with surgery, showing 5-year tumor control rates ranging from 86 to 99% [5–7]. Moreover, the effectiveness of SRS as primary therapy has also been reported recently [8, 9].

Approximately 80–90% of meningiomas are classified as WHO grade 1 and are considered benign; however, a subset may recur and, in some cases, exhibit histopathological progression to a higher WHO grade, acquiring more aggressive clinical behavior [10–12]. This progression is thought to follow a stepwise biological process involving molecular alterations [13, 14]. Recent advances in multi-omics profiling have enabled refined molecular classification, which may better predict prognosis than the current WHO grading system [15–18]; however, the molecular mechanisms underlying such progression, particularly following SRS, remain poorly understood.

In this report, we describe a patient who developed marginal progression of meningioma after primary SRS. Stable and marginally progressed tumor components were separately analyzed using histopathological and molecular approaches. Comparison of these distinct tumor components provides new insights into the potential molecular mechanisms underlying WHO grade progression after SRS.

## Clinical summary

**Fig. 1:**
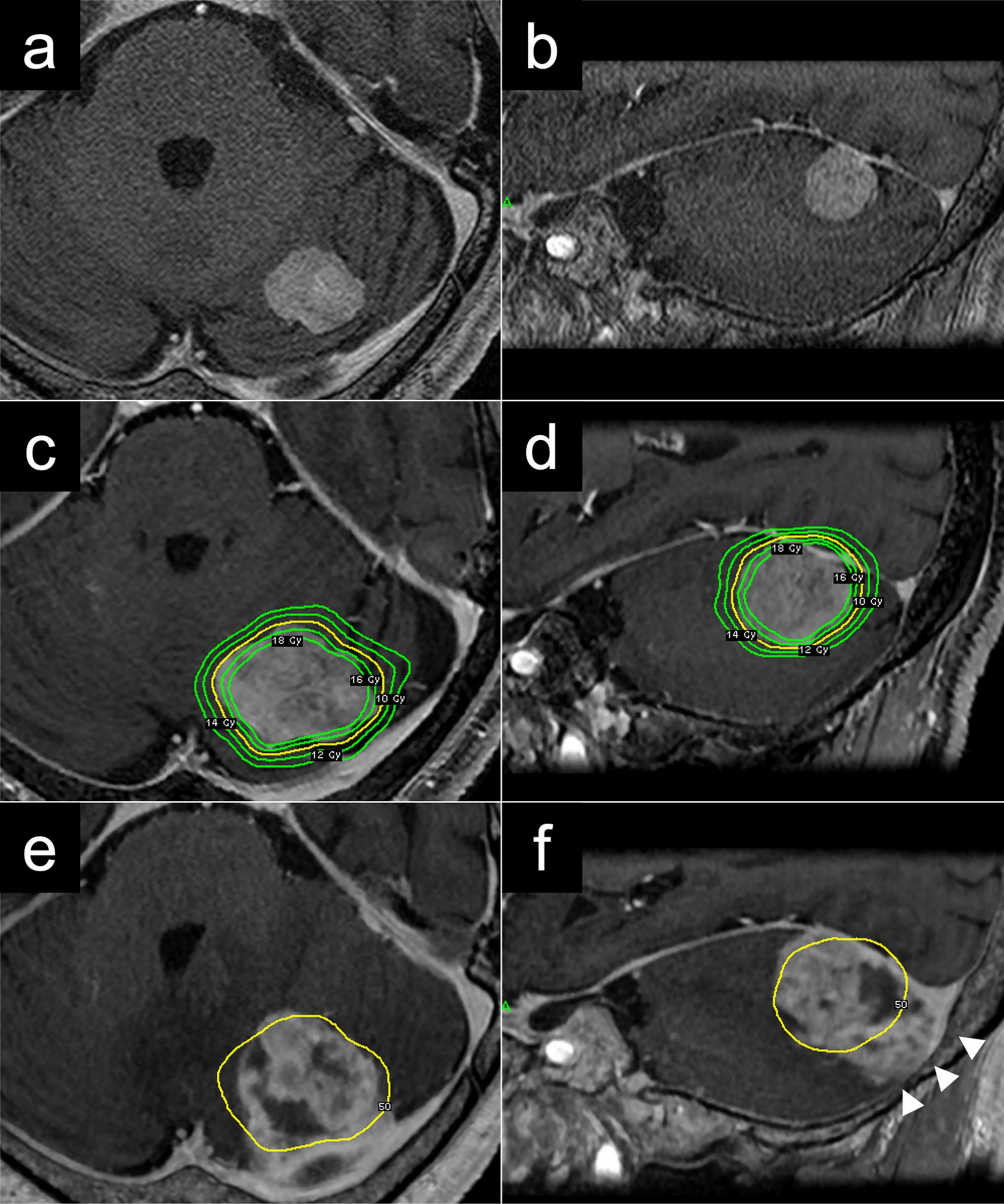
Gadolinium-enhanced T1-weighted magnetic resonance imaging at initial presentation, before, and after stereotactic radiosurgery (SRS). Axial and sagittal images at initial presentation show a 17-mm enhancing tumor at the cerebellar tentorium (**a**, **b**). Over 16 years, the tumor gradually grew to 33 mm by age 70. SRS was performed with a prescription dose of 14 Gy prescribed to the 50% isodose line (**c**, **d**). Follow-up images obtained 37 months after SRS show the controlled anterior irradiated tumor (yellow circle, 14 Gy to the 50% isodose line) and a newly developed posterior lesion (white arrowhead) (**e**, **f**)

**Fig. 2:**
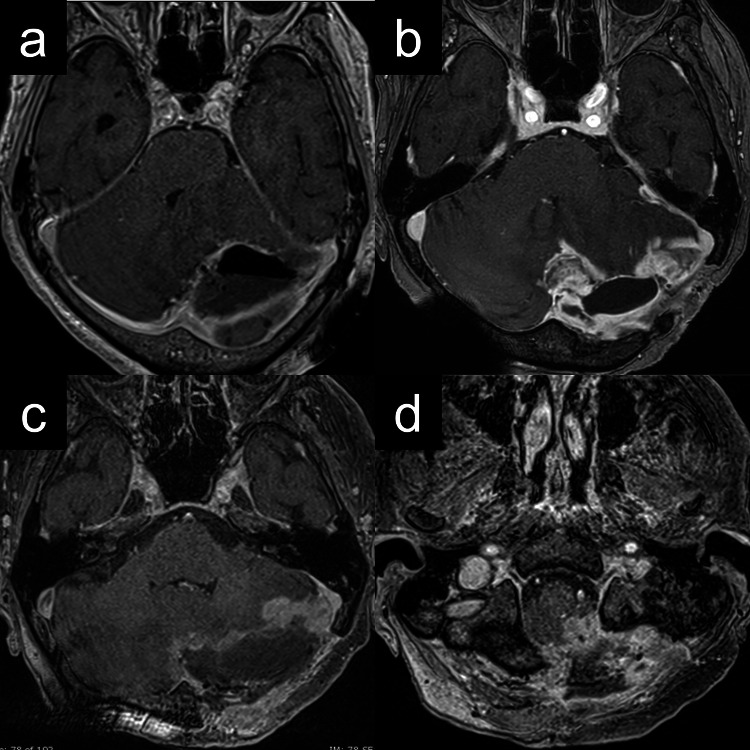
Clinical course of tentorial meningioma after initial resection on gadolinium-enhanced T1-Weighted magnetic resonance imaging (MRI). Tumor resection with suboccipital craniotomy was performed, achieving Simpson grade II resection (**a**). Three months later, MRI revealed massive tumor recurrence within the resection cavity (**b**). Although a second resection was performed, tumor progressed in the resection cavity and around the foramen magnum, suggestive of dissemination (**c**, **d**)

**Fig. 3:**
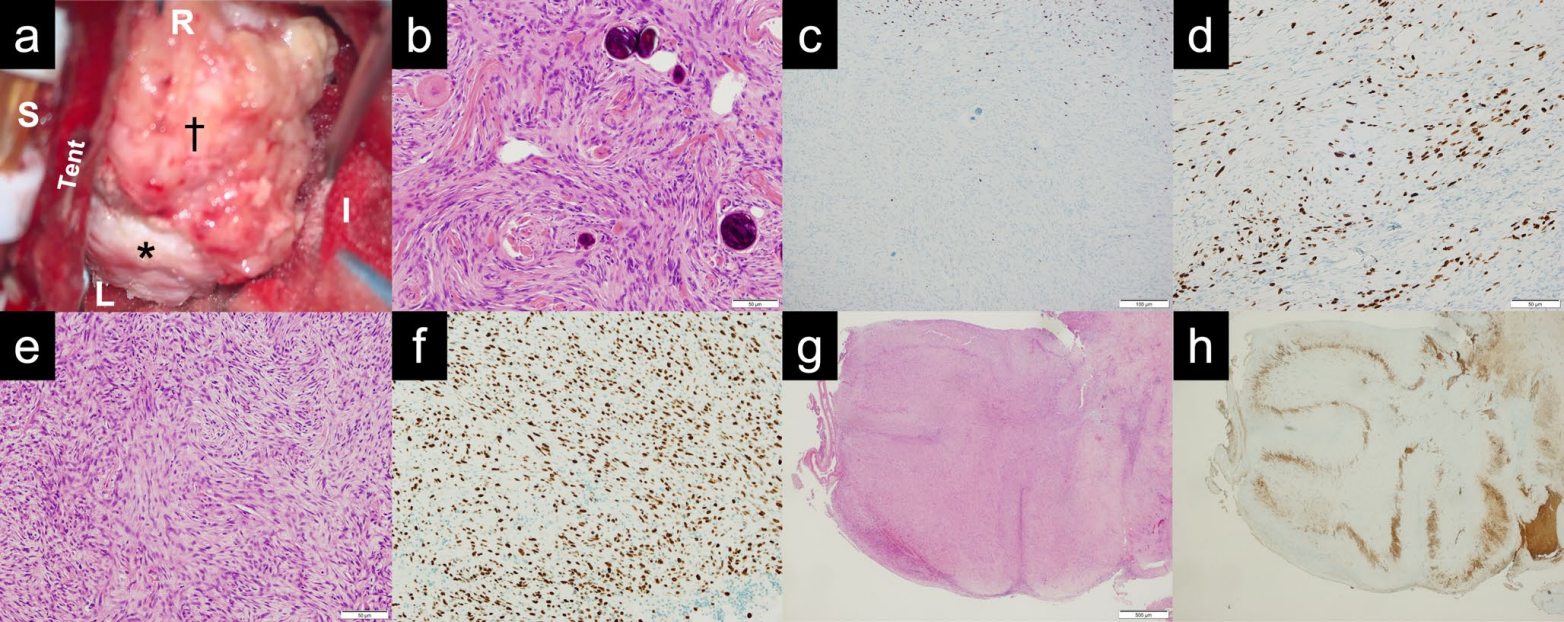
Intraoperative and histopathological images. **a**: Surgical image showing resection of a tentorial meningioma, including the anterior component with well-defined margins (*) and the posterior component, which was irregular in shape and invading the cerebellum (†). Abbreviations: R, right; L, left; S, superior; I, inferior. **b**: The anterior lesion, diagnosed as fibrous meningioma, showed spindle cells with whorled architecture and thick collagen fibers (hematoxylin and eosin; original magnification × 200; scale bar = 50 μm). **c**: The anterior lesion showed a Ki-67 labeling index mostly in the 1–4% range, showing some heterogeneity (original magnification × 100; scale bar = 100 μm). **d**: The anterior lesion showed a Ki-67 labeling index with up to 20% in the hot-spot regions. However, the diagnostic criteria for atypical meningioma were not met, leading to a diagnosis of WHO grade 1 fibrous meningioma (original magnification × 200; scale bar = 50 μm). **e**: The posterior lesion showed spindle cells with irregular, hyperchromatic nuclei, high mitotic activity, necrosis, and cerebellar invasion, consistent with anaplastic meningioma (hematoxylin and eosin; original magnification × 200; scale bar = 50 μm). **f**: The posterior lesion showed a Ki-67 labeling index > 50% (original magnification × 200). **g**, **h**: Hematoxylin and eosin staining showed that the tumor diffusely infiltrated the cerebellum (original magnification ×20), and GFAP staining demonstrated preserved cerebellar architecture within the tumor (original magnification ×20)

A 54-year-old woman was incidentally found to have a 17-mm tentorial meningioma on magnetic resonance imaging (MRI) (Fig. 1a, b). The tumor was initially followed with serial imaging, but a temporary interruption in outpatient follow-up delayed timely intervention. Upon re-evaluation at the age of 70, the lesion measured 33 mm. As the patient opted for a less invasive treatment rather than open surgery, primary SRS was performed, delivering a prescription dose of 14 Gy to the 50% isodose line (Fig. 1c, d). The treated lesion remained radiologically stable for 30 months after SRS, accompanied by preserved neurological function and an overall favorable clinical course. At the age of 73, she developed truncal ataxia and nausea. MRI showed that the originally irradiated tumor remained stable; however, a rapidly enlarging marginal progression was detected just posterior to the original tumor site over a 7-month period, accompanied by peritumoral edema and hydrocephalus (Fig. 1e, f). Tumor resection with suboccipital craniotomy was performed, achieving Simpson grade II resection (Fig. 2a). Intraoperatively, the anterior component with well-defined margins was clearly distinguished from the posterior component, which was irregular in shape and invading the cerebellar parenchyma (Fig. 3a). A ventriculoperitoneal shunt was subsequently placed for hydrocephalus. Three months after the initial resection, MRI revealed massive tumor recurrence within the resection cavity (Fig. 2b). Salvage hypofractionated SRS was delivered in five fractions, with a total prescription dose of 32 Gy to the 50% isodose line. However, further progression occurred in the resection cavity and around the foramen magnum, suggestive of dissemination, and was accompanied by severe headache, vomiting, and disturbed consciousness. Despite a second resection aimed at reducing brainstem compression to relieve symptoms, tumor control was ultimately not achieved (Fig. 2c, d). She succumbed 46 months after the initial SRS and only 8 months after the marginal progression.

### Pathological findings

The initial pathological examinations distinguished between the anterior component (irradiated and stable) and the posterior component (rapidly enlarging marginal progression). The anterior lesion showed spindle-shaped cells with whorled architecture and thick collagen fibers (Fig. 3b). Ki-67 labeling index was mostly in the 1–4% range, showing some heterogeneity (Fig. 3c), with up to 20% in the hot-spot regions (Fig. 3d). Some areas showed slightly smaller tumor cells with mildly increased cellularity; however, no other features of atypical meningioma, such as increased mitotic activity, brain invasion, prominent nucleoli, sheet-like growth pattern, or necrosis, were observed. Therefore, the diagnostic criteria for atypical meningioma were not met, leading to a diagnosis of WHO grade 1 fibrous meningioma throughout the entire lesions. In contrast, the posterior lesion consisted of spindle cells with irregular, hyperchromatic nuclei, high mitotic activity (10/10 HPF, 5 mitoses/mm², field area: 0.55 mm²), necrosis (Fig. 3e), a Ki-67 labeling index exceeding 50% (Fig. 3f), and tumor infiltration into the cerebellar architecture (Fig. 3g, h). It lacked typical meningioma features and exhibited sarcomatous characteristics, consistent with WHO grade 3 anaplastic meningioma. At the second craniotomy, histopathology reconfirmed anaplastic meningioma, with a Ki-67 labeling index exceeding 70% and 24 mitoses per 10 HPF (12 mitoses/mm², field area: 0.55 mm²).

**Fig. 4:**
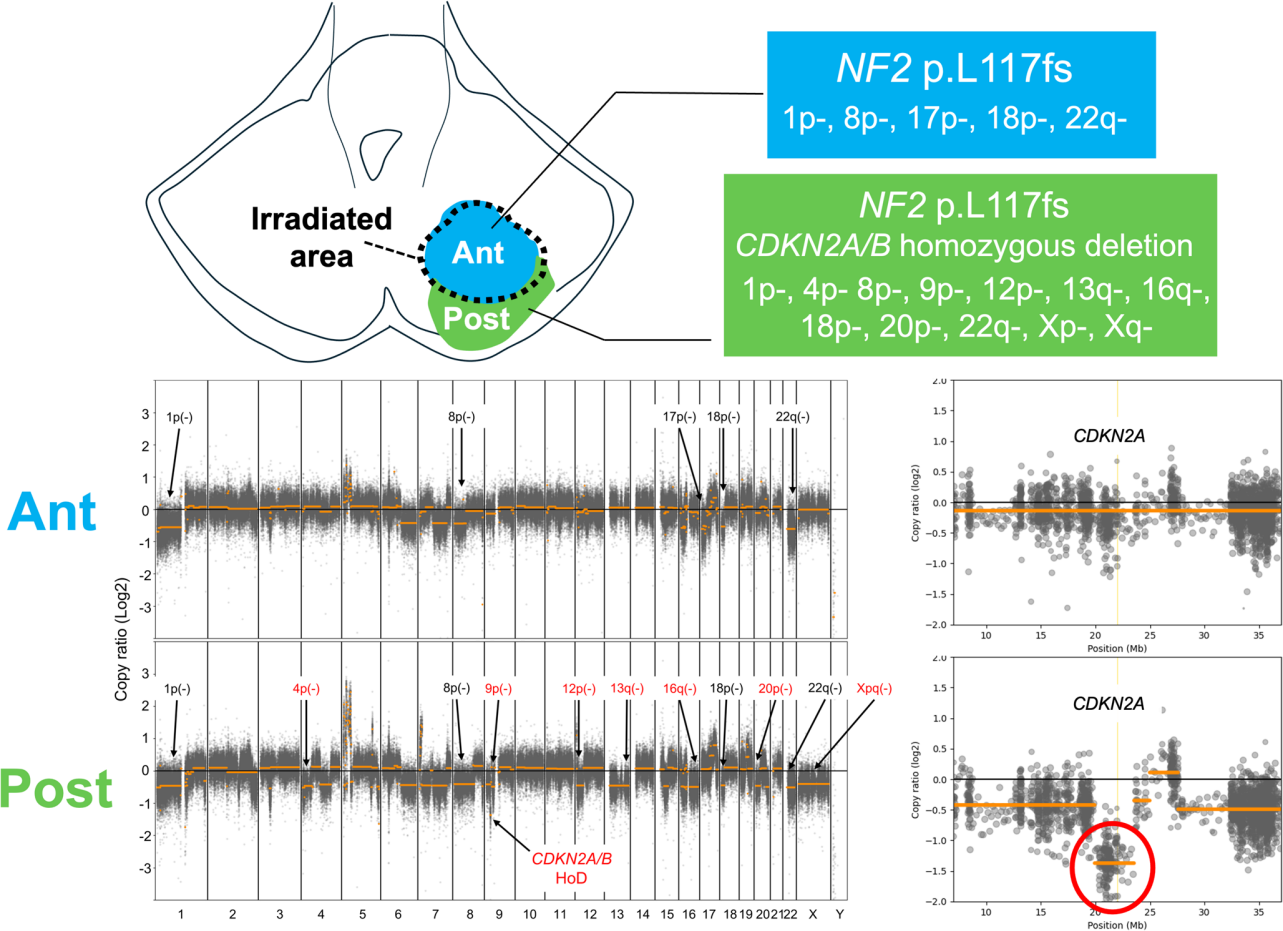
Comparative molecular profiles of the anterior and posterior tumor components. Both components shared an *NF2* (p.L117fs) mutation and deletions of 1p, 8p, and 18p. The posterior component showed additional CNAs, including deletions at 4p, 9p, 12p, 13q, 16q, 20p, Xp, and Xq. A log2 ratio below − 1.1 for *CDKN2A/B* indicated homozygous deletion

Whole-exome sequencing was performed on frozen tissue samples obtained separately from the anterior and posterior tumor components (Fig. 4), as described previously [19, 20]. The anterior component harbored an *NF2* (p.L117fs) pathogenic mutation and copy number alterations (CNAs) involving losses of 1p, 8p, 17p, and 18p. Because of the presence of 1p and 22q losses, the anterior component is molecularly assigned to WHO grade 2 according to cIMPACT-NOW update 8 [21].The posterior component shared *NF2* (p.L117fs) mutation but exhibited a broader range of CNAs, including deletions at 1p, 4p, 8p, 9p, 12p, 13q, 16q, 18p, 20p, 22q, Xp, and Xq. Notably, the log2 ratio for *CDKN2A/B* in the posterior component was below − 1.1, suggestive of homozygous deletion [22].

## Discussion

In this case, the stable lesion, diagnosed as a fibrous meningioma, and the marginally progressed lesion, diagnosed as an anaplastic meningioma, were pathologically and clinically distinct components within the same tumor. Additional CNAs associated with poor prognosis, such as homozygous deletion of *CDKN2A/B* and whole X chromosome loss, were identified exclusively in the marginally progressed component. Homozygous deletion of *CDKN2A/B* is a marker of poor prognosis [23, 24] and the most frequent genomic alteration linked to WHO grade progression [25]. Similarly, X chromosome loss correlates with shorter progression-free survival [26]. While previous studies have reported intratumoral heterogeneity in progressive meningiomas [27], this is the rare case of pathologically and molecularly distinct components within a single tumor after SRS.

WHO grade progression during the clinical course is a rare phenomenon; approximately 2–4% of benign meningiomas progress to higher-grade tumors [11, 12, 28], and 17–38% of atypical meningioma and 54–70% of anaplastic meningiomas are derived from lower-grade tumors [29–31]. Stepwise genetic alterations have been proposed as a mechanism underlying this progression, with *NF2* gene inactivation recognized as an early event followed by additional chromosomal deletions of 1p, 14q, and 10q, ultimately leading to more aggressive phenotypes [13, 14, 32]. Although sequential evaluations were not performed, this case likely represents spatial heterogeneity reflecting WHO grade progression. Because only whole-exome sequencing was performed in the present case, complete classification was not possible; however, based on recent multi-omics classifications, the anterior and posterior components corresponded to MG3 (hypermetabolic) and MG4 (proliferative) according to Nassiri et al. [18], and to methylation classes intermediate-A and intermediate-B according to Sahm et al. [33]. Both were categorized as hypermitotic by Choudhury et al. [15]. Both tumor components shared *NF2* inactivation and 1p loss, suggesting that histologically benign tumor cells may harbor malignant potential and subsequently acquire additional alterations driving aggressive behavior [34, 35].

The relationship between SRS and pathological progression remains poorly understood and controversial. In the long-term follow-up studies, pathological progression after SRS is rare, with reported rates of 0–2.2% [36–38] A systematic review and meta-analysis resulted in no definitive evidence that SRS induces pathological progression [39]. In contrast, rare individual reports have suggested that SRS may induce the progression of meningiomas to higher-grade lesions, including cases with histopathological features of osteosarcoma and chondrosarcoma [40–42]. Furthermore, meningiomas previously treated with adjuvant radiation therapy show a higher frequency of CNAs compared to radiation-naïve tumors [43], and radiation-induced malignancy may exhibit sarcomatous features [44, 45]. In our case, the progressed lesion demonstrated anaplastic histology with sarcomatous features and additional CNAs related to malignancy. These findings raise the possibility that SRS may have contributed to pathological progression; however, given the absence of pre-SRS pathological and molecular data, spontaneous WHO grade progression cannot be excluded. Therefore, it remains uncertain whether SRS contributed to pathological progression, and further investigation in larger cohorts is warranted.

A limitation of this study is that the boundary between the anterior and posterior components could not be evaluated, as the specimens were submitted separately. Therefore, the morphological continuity between the two components remains uncertain. Another limitation is the lack of pre-SRS pathological and molecular data, which makes it difficult to determine the relationship between SRS and tumor progression.

## Declarations

### Previous presentations

This work has not been previously presented.
