# Correction: Comparative molecular profiles of distinct tumor components in recurrent tentorial meningioma after stereotactic radiosurgery: a case report implicating acquired aggressive alterations associated with WHO grade progression

**DOI:** 10.1007/s10014-026-00535-5

**Published:** 2026-04-05

**Authors:** Takeru Hirata, Yudai Hirano, Motoyuki Umekawa, Satoru Miyawaki, Yuki Shinya, Hirotaka Hasegawa, Yu Sakai, Noritaka Kudo, Daisuke Komura, Hiroto Katoh, Shumpei Ishikawa, Nobuhito Saito

**Affiliations:** 1 Department of Neurosurgery, The University of Tokyo Hospital, 7-3-1 Hongo, Bunkyo-ku, Tokyo, 113-8655 Japan; 2 Department of Neurosurgery, University of Virginia, Charlottesville, VA USA; 3 Department of Pathology, The University of Tokyo Hospital, Bunkyo-ku, Tokyo, Japan; 4 Department of Preventive Medicine, Graduate School of Medicine, The University of Tokyo, Bunkyo-ku, Tokyo, Japan

**Correction: Brain Tumor Pathology**

10.1007/s10014-025-00524-0

In this article, the ‘Ethical standards’ statement was incorrectly given as ‘This study was approved by the Clinical Research Review Board of The University of Tokyo (approval no. 2231) and conducted in accordance with the Declaration of Helsinki. Written in formed consent for publication was obtained from the patient.' but should have been 'This study was approved by the Clinical Research Review Board of The University of Tokyo (approval no. 2231 and G10028) and conducted in accordance with the Declaration of Helsinki. Written informed consent for publication was obtained from the patient.’

The original article has been corrected.

